# Considerations for the assessment of blast exposure in service members and veterans

**DOI:** 10.3389/fneur.2024.1383710

**Published:** 2024-04-15

**Authors:** Jared A. Rowland, Sarah L. Martindale

**Affiliations:** ^1^Salisbury VA Healthcare System, Salisbury, NC, United States; ^2^Veterans Integrated Service Network (VISN)-6 Mid-Atlantic Mental Illness, Research Education and Clinical Center (MIRECC), Durham, NC, United States; ^3^Wake Forest School of Medicine, Winston-Salem, NC, United States

**Keywords:** blast (explosion) wave-induced neurotrauma, veteran, traumatic brain injury, neurotrauma, assessment

## Abstract

**Introduction:**

Blast exposure is an increasingly present occupational hazard for military service members, particularly in modern warfare scenarios. The study of blast exposure in humans is limited by the lack of a consensus definition for blast exposure and considerable variability in measurement. Research has clearly demonstrated a robust and reliable effect of blast exposure on brain structure and function in the absence of other injury mechanisms. However, the exact mechanisms underlying these outcomes remain unclear. Despite clear contributions from preclinical studies, this knowledge has been slow to translate to clinical applications. The present manuscript empirically demonstrates the consequences of variability in measurement and definition across studies through a re-analysis of previously published data from the Chronic Effects of Neurotrauma Study 34.

**Methods:**

Definitions of blast exposure used in prior work were examined including Blast TBI, Primary Blast TBI, Pressure Severity, Distance, and Frequency of Exposure. Outcomes included both symptom report and cognitive testing.

**Results:**

Results demonstrate significant differences in outcomes based on the definition of blast exposure used. In some cases the same definition was strongly related to one type of outcome, but unrelated to another.

**Discussion:**

The implications of these results for the study of blast exposure are discussed and potential actions to address the major limitations in the field are recommended. These include the development of a consensus definition of blast exposure, further refinement of the assessment of blast exposure, continued work to identify relevant mechanisms leading to long-term negative outcomes in humans, and improved education efforts.

## Background

The long-term consequences of exposure to blasts have become an increasing concern among the military population. Although mild traumatic brain injury (TBI) is considered the signature injury of the recent wars in Iraq and Afghanistan, many more service members have been exposed to blasts during this time. Most TBIs that occur in combat zones involve a blast, yet incongruently, blast exposure does not typically result in TBI (1–4). Since the onset of these conflicts, our understanding of the effects of TBI and how to treat them has advanced significantly; however, far less progress has been made in understanding and treating the effects of blast exposure (with or without resulting TBI). Additionally, there are several definitions of TBI that are widely accepted, yet no definition for blast exposure exists (5–7). The purpose of this manuscript is to present the current challenges in the study of blast exposure related specifically to variability in definitions and assessment approaches for blast exposure history. We initially provide context for the current challenges to the field and then present examples of how variability in the definition of blast exposure can alter study conclusions. Finally, we propose steps to address these challenges including a widely endorsed definition of blast exposure, continued mechanistic work to improve our understanding of blast exposure, education regarding the effects of blast exposure, and development of improved assessments of blast exposure.

## Blast as a mechanism of neurotrauma

At present, there is little debate that blast overpressure independently affects the brain (8–11). Two complimentary lines of evidence strongly support that primary blast forces (i.e., the direct effect of the blast wave) interact with and alter brain structure and function in the absence of other mechanisms. First, preclinical studies have incontrovertibly demonstrated that primary blast forces can directly affect brain structure and function in the absence of blunt force and other non-blast mechanisms (12, 13). Consistent findings have been reported across several species including mice, rats, ferrets, pigs, and non-human primates, suggesting that the mechanism through which blast interacts with brain tissue is robust across species (12, 14–19). Over the past decade, preclinical models have evaluated low-level blast that does not create head movement, further clarifying that blast-related injuries are induced directly through properties of the blast wave rather than other mechanisms such as head motion (20, 21). More recent work has applied an open-air blast model to better reflect environmental characteristics consistent with military deployment scenarios (22). These results clearly establish aspects of the blast wave as a mechanism that can directly induce changes to the brain in the absence of other injury mechanisms.

Second, studies of breachers (i.e., individuals participating in training programs for the use of explosives) offer additional support for the blast wave as a primary mechanism. Results indicate differences between career breachers and demographically-matched controls in brain structure and function (23), gene expression (1), and blood-based markers (2). Other work has examined individuals before and after explosives training, demonstrating effects of exposure to low-level blast on cognition (3, 24), as well as brain structure (25) and function (23). Results of simulation studies offer further insight into the potential mechanisms of this effect and how they may differ from those related to blunt force impacts (26, 27). When considering results across preclinical and human studies, it is evident that primary blast forces directly interact with brain tissue in the absence of other mechanical forces. Further, it is clear that exposure to primary blast forces can result in the alteration brain function and structure under these circumstances.

Blast exposure has been associated with a wide range of negative outcomes in human studies including alterations in brain structure (23, 28–31) and function (23, 32–34), poorer cognitive functioning (3, 35), as well as increased psychiatric and health symptom severity (36, 37). These studies have established this relationship beyond the effects of PTSD, TBI, and other covariates. As noted previously, blast exposure can serve as a mechanism that induces TBI, similar to other established mechanisms such as blunt force impact and acceleration/deceleration, among others (4). The evidence for significant negative outcomes following exposure to blast is compelling, especially when considered in the context of findings from preclinical work (38–41). Although the relationship between blast and negative outcomes is robust across studies, the methods used to assess, characterize, and define blast exposure are inconsistent and highly variable.

## Defining blast exposure

Comparison of results across studies to determine robust mechanisms through which blast exposure influences long-term outcomes is limited by inconsistent methodological approaches. A significant factor influencing this inconsistency is the variability in definitions and measurement of blast exposure, considering the notable range in the frequency and severity of exposures (14, 15). Service members are exposed to different types of blast overpressure throughout their military career across different environments (i.e., in garrison, deployment) and contexts (i.e., training, combat, outgoing, incoming). Further, these exposures can be concussive (i.e., causing symptoms congruent with a TBI) or subconcussive. This variability in exposure ultimately forms a complex taxonomy that has hindered the development and standardization of definitions and methods used to measure blast across research studies.

A wide range of definitions for blast exposure have been utilized in published work including the distance from the source (32, 42, 43), reported severity of blast overpressure (31, 37), any report of exposure to blast overpressure (28), report of physical effects of blast overpressure (44), using military occupational specialty (MOS) to categorize blast risk (45), or objective data from wearable monitors (24). Each of these approaches has contributed to our understanding of the acute and chronic effects of blast exposure. However, there are considerable differences among these approaches that limit our ability to draw meaningful conclusions. As a result, it is not possible to synthesize these study outcomes into a coherent understanding of the effects of blast exposure on the brain, the underlying mechanisms, or the resulting consequences.

## Assessment of blast exposure

Definitions of blast exposure have been operationalized using a variety of assessment measures. These include dedicated TBI evaluations, dedicated blast evaluations, *ad hoc* items to evaluate blast exposure, and body mounted sensors using various calculated outcomes. Many of these measures have been reviewed elsewhere; however, there are several key points that warrant restatement (46). First, TBI interviews typically include an evaluation of the injury mechanism, with blast being just one of many. Several interviews have specific questions to evaluate concussive events that involve blast, and some have added additional questions to evaluate the frequency of these events (47, 48). However, blast exposure does not always result in symptoms of concussion or mild TBI. TBI evaluations are not designed or intended to assess non-concussive events; therefore, they will likely underestimate the frequency and severity of these events. As noted previously, clinical symptoms of TBI are not necessary for blast-induced neurotrauma to occur. This means that these measures may miss important blast events that could have a significant influence on future outcomes. In addition, these measures are validated to identify and diagnose TBI. There is generally no additional psychometric work conducted to evaluate the reliability or validity of the blast-specific aspects of these measures. Overall, these measures are excellent at identifying blast-related TBI but may overlook important non-concussive blast events. Further, these measures do not collect detailed information about characteristics of non-concussive blast exposures (e.g., pressure experienced, protective factors present) that have shown to be associated with acute and chronic effects following blast exposure.

Dedicated evaluations of blast offer a more comprehensive characterization of blast exposure than TBI interviews, specifically because they capture events that are not considered potentially concussive (46). Most available measures evaluate the frequency of different types of blast exposures. However, significant variability among these measures remains, mostly due to the difficulty capturing the severity of the blast exposure, even among similar events. Because of this, there are no standard outcomes across these assessment tools. Additionally, categories of events can overlap across measures making direct comparisons difficult. However, these measures offer the most nuanced evaluation of an individual’s blast exposure history.

*Ad hoc* items evaluating blast exposure are frequently utilized as part of survey measures (49, 50). These are typically one or two self-reported items asking about the frequency of a certain kind of exposure (e.g., “In your life, how many times have you been close enough to an explosion in which you felt the blast wave?” (49, 51)). There is high variability among this category of measurement and the reliability and validity of these measures has typically not been evaluated. Of note, proxy measures for blast are also frequently used, especially for retrospective chart review studies, such as MOS (45, 52).

Finally, body mounted sensors are unique among blast assessments as they are an objective measurement of the blast intensity (3, 53–55). Efforts are ongoing to determine the ideal method to deploy these sensors and analyze the resulting data (56, 57). Although objective measurement of a blast is ideal to assess resulting effects, individual variability still exists (e.g., sensor location relative to direction of the blast, placement of sensors, number of sensors) and individuals are not wearing sensors on a consistent basis. Therefore, sensors represent a strong method for directly measuring characteristics of a blast; however, they are unlikely to provide a comprehensive account of an individual’s exposure to blasts when used in isolation.

Currently, no broadly accepted standards exist to determine when a blast is likely to result in significant sequelae or require treatment or intervention. Blasts are defined as either concussive or subconcussive, using criteria developed to diagnose and identify TBI. As a result, blast exposures are evaluated with standards largely based on non-blast mechanisms of brain injury. It is critical to have a clear operational definition of a condition in order to successfully identify, study, and treat that condition. In the absence of well-defined criteria, the study of blast exposure will continue to be restricted by project-specific definitions. There is a critical need to understand the blast characteristics and their respective thresholds that are associated with negative outcomes to support the development of a consistent and clear definition of blast exposure, much like those developed and implemented for TBI.

## Demonstration of pitfalls due to measurement inconsistency

To illustrate these points and the broad issue of variability in measurement, we present new analyses of previous work (35, 37) that provide a side-by-side comparison of results when using different definitions of blast exposure identified in published human studies, including: blast TBI (34, 58), primary blast TBI (28), pressure severity (4, 37), distance from the blast (32, 43), and frequency of exposure (49–51). These analyses utilize data from the Chronic Effects of Neurotrauma Consortium Study 34 (CENC-34) that has been described previously (35, 37). Briefly, inclusion criteria for CENC-34 were: age 18 years or older, deployed in support of the wars in Iraq and Afghanistan, and reported exposure to combat. Exclusion criteria were: history of moderate to severe TBI or penetrating head injury, non-deployment TBI with loss of consciousness, history of major neurologic disorder, history of serious mental illness, current neurocognitive disorder, substance use disorder, or psychosis. Participants completed questionnaires, structured clinical interviews, cognitive testing, and neuroimaging.

Samples for analyses are from two published manuscripts using CENC-34 data evaluating effects of blast exposure on symptom report (37) and cognitive function (35). Participants included in symptom analyses (*N* = 275) passed symptom validity measures and participants in cognitive analyses (*N* = 254) passed cognitive validity measures. Current analyses parallel those presented in the published work, including covariates. Time variables (i.e., time since most recent injury, first injury, most recent blast exposure, first blast exposure, first traumatic event, deployment-related traumatic event, most recent traumatic event, and deployment) were considered as covariates but not significantly associated with other variables in bivariate models, did not improve models, and did not have significant independent effects and were therefore not included in final models. We have re-analyzed the data to demonstrate the effect of different definitions of blast exposure: Blast TBI, Primary Blast TBI, Pressure Severity, Distance (≤3 meters and ≤10 meters), and Frequency of Exposures. Operational definitions of these variables are detailed in Table 1. Definitions are based on data from the Mid-Atlantic MIRECC Assessment of TBI [MMA-TBI (59)] and the Salisbury Blast Interview [SBI (4)]. The MMA-TBI is a well-validated, semi-structured TBI interview that determines the presence or absence of TBI based on VA/DOD guidelines. Information regarding the potential concussive mechanisms present, including blast, are also collected. The SBI is a semi-structured interview that characterizes blast exposure across the lifespan regardless of the presence of TBI. By evaluating effects from a blast itself and not symptoms specific to TBI diagnosis, the SBI provides a comprehensive evaluation of blast exposure, including subconcussive exposures such as heavy weapons training, smaller detonations, and blast events at a further distance. Analyses were conducted using linear regression. Dependent variables were self-reported symptom measures scores or demographically-adjusted outcomes of cognitive testing. Each model included one of the blast variables from Table 1, presence or absence of PTSD diagnosis from the Clinician Administered PTSD Scale-5 [CAPS-5 (60)], and other covariates identified previously.

**Table 1:** Operational definitions of blast variables.

Variable	Data source	Type	Operational definition	Symptom sample	Cognitive sample
Blast TBI	MMA-TBI	Dichotomous	TBI with blast present as one potential concussive mechanism. This includes TBI with mixed blast and blunt mechanisms	*n* = 95	*n* = 85
Primary blast TBI	MMA-TBI	Dichotomous	Presence of at least one TBI for which blast was the only mechanism present. May include individuals with additional history of blunt TBI	*n* = 76	*n* = 68
Pressure severity	SBI	Continuous	Maximum reported severity of exposure to a pressure wave. The experienced pressure gradient of each blast event is rated by a participant on a given a behaviorally anchored Likert scale	Mean = 1.8 SD = 1.5	Mean = 1.9 SD = 1.5
Distance	SBI	Dichotomous	Distance ≤3 meters and ≤10 meters. This variable is positive if the participant reported any blast exposure below the noted threshold	3 meters *n* = 65 10 meters *n* = 111	3 meters *n* = 62 10 meters *n* = 105
Frequency	SBI	Continuous	Total lifetime number of reported exposures to blast, defined as experiencing any pressure gradient, defined as a score >0 on the SBI pressure scale	Mean = 169.3 SD = 575.4	Mean = 182.9 SD = 593.7

TBI, traumatic brain injury; MMA-TBI, Mid-Atlantic MIRECC Assessment of TBI; SBI, Salisbury Blast Interview.All TBI in this sample were mild in severity.

The significant effects of specific blast definitions on symptom outcomes and quality of life are reported in Table 2. These results illustrate highly variable outcomes based on the definition of blast exposure that is used. Depending on the specific definition, outcomes ranged from no significant associations across all outcomes for frequency and distance characteristics, to a significant association with almost all symptom measures for the experienced pressure severity. Variables based on the TBI interview had mixed effects, as evidenced by significant associations with neurobehavioral symptoms and quality of life, but not other variables.

**Table 2:** Parameter estimates of the effect for various definitions of blast exposure on symptom presentation.

	Blast TBI	Primary blast TBI	Pressure severity	Distance		Frequency
				≤3 meters	≤10 meters	
PCL-5 total	ns	ns	2.38^*^	ns	ns	ns
PHQ-9 total	ns	ns	0.92^*^	ns	ns	ns
NSI total	4.83^*^	4.79^*^	2.15^*^	ns	ns	ns
PSQI global	ns	ns	0.42^*^	ns	ns	ns
PROMIS PI T	ns	ns	ns	ns	ns	ns
QOLIBRI total	−9.75^*^	−8.43^*^	−2.64^*^	ns	ns	ns

*N* = 275, ns, non-significant; ^*^p < 0.05, all models adjust for PTSD diagnosis and DRRI-2 combat exposure, significance unadjusted for multiple comparisons; PCL-5, PTSD Checklist-5; PHQ-9, Patient Health Questionnaire-9; NSI, Neurobehavioral Symptoms Inventory; PSQI, Pittsburgh Sleep Quality Inventory; PROMIS PI, PROMIS Pain Interference Short Form T-Score; QOLIBRI, Quality of Life After Brain Injury.

The effect of different blast exposure definitions on cognitive outcomes can be seen in Table 3. As seen with symptoms outcomes, a wide range of outcomes are present. Very close exposure (≤3 meters) was significantly associated with the majority of cognitive outcomes; however, exposure within ≤10 meters (inclusive of exposures ≤3 meters) was only significantly associated with a single outcome. Frequency of exposure was associated with several non-processing speed measures, whereas experienced pressure severity was not significantly associated with any outcome. TBI-based definitions were only associated with Trail Making Test (TMT) Part B performance.

**Table 3:** Parameter estimates of the effect for various definitions of blast exposure on cognitive testing.

	Blast TBI	Primary blast TBI	Pressure severity	Distance		Frequency
				≤3 meters	≤10 meters	
WAIS-IV FSI	ns	ns	ns	−3.79^*^	ns	−0.002^*^
WAIS-IV VCI	ns	ns	ns	−3.51^*^	ns	−0.003^*^
WAIS-IV PRI	ns	ns	ns	−3.04^*^	ns	−0.003^*^
WAIS-IV WMI	ns	ns	ns	ns	ns	ns
WAIS-IV PSI	ns	ns	ns	ns	ns	ns
TMT Part A	−4.55^*^	−3.94^*^	ns	−4.23^*^	ns	ns
TMT Part B	ns	ns	ns	−3.91^*^	−2.79^*^	ns

*N* = 254, ns, non-significant; ^*^p < 0.05, all models adjust for PTSD diagnosis, significance unadjusted for multiple comparisons, *t*-scores adjusted for age, education, sex, and race; WAIS, Wechsler Adult Intelligence Scale; FSI, Full Scale Index; VCI, Verbal Comprehension Index; PRI, Perceptual Reasoning Index; WMI, Working Memory Index; PSI, Processing Speed Index; TMT, Trail Making Test.

There are clear differences in how each definition is associated with either symptom or cognitive outcomes. Distance and frequency measures were associated with cognitive outcomes, but not symptom outcomes. Within distance variables, the sensitivity to outcomes differed largely by the selected distance. Experienced pressure severity was strongly associated with symptom outcomes, but not cognitive outcomes. TBI variables were not strongly associated with cognitive outcomes but there were indicated relationships with some symptom outcomes. It should be noted that all TBI were mild in severity; therefore, broad impairments in outcomes are not anticipated for large numbers of participants. In addition, participants were seen a decade post-deployment on average; therefore, symptoms do not represent acute effects of TBI or blast exposure and are considered long-term outcomes.

These analyses demonstrate how the definition of blast exposure can dramatically affect the outcome of a study. If the current results were acquired across different studies, it would not be possible to synthesize the outcomes into a coherent understanding of the consequences of blast exposure. At best, it could be possible to conclude that some forms of blast exposure potentially have negative consequences. However, we would be left to speculate as to the reason that one definition was highly relevant and another was not.

Because the outcomes presented here are from the same sample, it is possible to further explore the nuances of these relationships. Based on these results, it is clear that blast exposure can result in negative outcomes even in the absence of symptoms consistent with TBI. Specifically, these results suggest that the effects of blast-induced TBI and the effects of blast exposure could be viewed as two separate fields of study. There was very little consistency in the pattern of relationships of blast TBI and blast characteristic variables to outcomes. This is consistent with work that has investigated blast-induced neurotrauma (BINT), the direct effects of blast on brain structure and function regardless of other circumstances or context (10, 16). BINT differs from blast-induced TBI as it does not rely on symptoms to infer that a neurological insult occurred. To be classified as TBI, an event must meet the clinical definition that includes alteration of consciousness or another neurological symptom occurring immediately following the traumatic force exerted on the central nervous system (e.g., blunt force impact, acceleration/deceleration, blast, etc.) (5). In contrast, BINT is evaluated following a blast exposure by looking for the outcome of interest (e.g., change in brain volume, alterations in brain function), regardless of symptoms at the time of the event. Consistent with prior work on BINT, the presented results demonstrate that clinical symptoms of TBI are not necessary for blast exposure to result in chronic alterations to brain structure and function, or long-term negative outcomes to occur (29, 31, 33, 49, 61, 62).

Using clinical definitions of TBI to determine if a particular blast exposure has clinical relevance will almost certainly result in false negative diagnosis and missed opportunities to provide treatment or intervention. The clinical diagnosis of TBI is largely based on evidence and experience from non-blast-induced events (5, 7, 63). This includes sports injuries, falls, motor vehicle accidents, assaults, etc. These events typically involve the direct transfer of forces to the central nervous system through contact with the skull or body (e.g., striking the head on an object). Blast exposure typically involves the indirect transfer of a force across distance (acknowledging the potential for acceleration/deceleration injuries) and is associated with a number of potential injury mechanisms (8–11). This creates the potential for blast events to result in a different acute symptom presentation from events involving non-blast forces, a scenario that could explain chronic post-concussive-like symptoms that have been frequently reported following non-concussive blast exposures. In summary, reliance on clinical definitions of TBI will continue to limit the development of an accurate and comprehensive understanding of how blast exposure affects long-term outcomes.

This study has limitations that should be considered, with several previously identified in the original manuscripts. Recall and attribution bias are inherent in all assessments of historical events. Participants were all combat exposed veterans, therefore results may not generalize to individuals without combat exposure, active duty service members, or civilians experiencing blast. In combat scenarios blast frequently co-occurs with other forces that may induce TBI (e.g., blunt force impact), it is possible outcomes following mixed force exposures and injuries are different from those involving a single force. Finally, individuals were on average over a decade beyond their most recent TBI or blast exposure. Results may be different in the acute phase following blast exposure. Notable strengths include the use of structured clinical interviews to assess psychiatric conditions and obtain TBI and blast exposure histories. Symptom and performance validity were evaluated using standalone measures.

## Potential actions to address limitations in the study of blast exposure

There are several potential actions to address the issues surrounding the study of blast exposure and blast-induced TBI that have been noted here. The most critical action is the development of a standard definition of blast exposure, similar to those that exist for TBI, the process for which is well-established and could be easily emulated. A standard definition would provide a clear point of comparison across studies, addressing the most prominent weakness in the study of blast exposure. Establishing a workgroup consensus opinion based on available empirical and clinical evidence would provide strong support and clear direction for the field. As demonstrated in this manuscript, the knowledge base to support a consensus definition of blast exposure is not fully developed. Therefore, this definition would need to be updated periodically as we learn new information and our understanding of blast exposure characteristics and outcomes expands.

Future research should incorporate any standardized definition of blast exposure alongside a full characterization of blast exposure. What constitutes a full characterization of blast exposure is a topic for workgroup discussion and consensus; however, this should likely include the date/time of the event, information about protective factors that were present, the distance from the munitions, and details about the severity of the blast (e.g., sensor data, ratings from the participant, or the munitions that detonated). This will allow a clear comparison of outcomes across studies as well as exploratory analyses to continue improving our understanding of how specific characteristics of events relate to outcomes. To support this, observational studies of human blast exposure measuring a wide range of blast characteristics are necessary. This will likely require the use of more than one available assessment of blast history. The results of these studies can then be combined with the results of well-controlled preclinical studies to translate preclinical mechanistic work into clinically applicable knowledge for initiating prevention and treatment efforts.

Related to the need for a standardized definition, the assessment of blast exposure can be significantly improved. Most human studies of blast exposure use data obtained from assessments of TBI. These measures were typically designed to diagnose TBI with blast being one of many potential mechanisms. This approach is limited because blast exposure does not always result in symptoms of concussion or TBI and clinical symptoms of TBI are not necessary for blast-induced neurotrauma to occur. Several interviews have added additional questions to evaluate the frequency of a certain type of blast event (e.g., within 10 meters, felt the blast wave). However, there has been no validation of these added blast event items. Therefore, it is critical that blast exposure be characterized using dedicated assessment measures that have been well-validated (46). The development and validation of blast measures is an area that will benefit from continued refinement to identify the characteristics of blast events most relevant to negative outcomes and the most robust method of measuring these characteristics.

Currently there are efforts by the Department of Defense to include data gathered by body mounted blast sensors to the medical record. This is a significant advancement to the characterization of blast exposure in service members and will allow this data to be analyzed along with medical record outcomes. A limitation of this approach is that service members are not always wearing sensors when they are exposed to blasts, especially in a combat theatre. Sensors may also malfunction and omit recording exposures. Therefore, it is important to administer other assessments of blast exposure in conjunction. Regularly administering a non-sensor assessment of blast exposure that would also be included in the medical record would provide an accessible second data point for analysis. Annual administrations could be completed as part of a yearly process. These two data sources would provide complementary information to improve the ability to characterize blast exposure and its relationship to outcomes.

Finally, there is a clear need for dissemination of knowledge about the effects of BINT. The empirical literature base clearly demonstrates that symptoms consistent with TBI are not necessary for BINT or negative outcomes to occur. However, many clinicians and researchers remain skeptical of this relationship. Concerted efforts are necessary to increase awareness about the potential for primary blast forces to directly induce neurotrauma and result in negative outcomes including psychiatric symptoms, cognitive problems, and reduced quality of life. These long-term effects likely represent not only the acute effects of blast exposure, but also chronic secondary “downstream” effects including neuroinflammation, neurotoxicity, cellular senescence, and neurodegeneration, among others (13, 64–67). Education at all levels (e.g., continuing education opportunities, round tables at annual conference meetings, grand rounds in hospital or academic medical center settings, journal clubs) will be required for both clinicians and researchers to improve awareness of primary blast forces as mechanisms of neurotrauma.

## Conclusion

The long-term negative consequences of blast exposure among service members and veterans have become increasingly evident since the start of the wars in Iraq and Afghanistan. However, our understanding of how and why blast exposure often results in negative consequences and the means to intervene or treat resulting symptoms and conditions has lagged comparatively. The absence of a broadly endorsed definition of blast exposure paired with the use of study-specific definitions prevent synthesis of existing literature into a cohesive understanding of the field. Preclinical work has undeniably demonstrated that primary blast forces can directly induce neurotrauma with associated, ongoing symptoms; however, these findings have not translated into clinical work. Collaborative efforts will be required to advance the study of blast exposure, including the development of a standardized definition of blast exposure, the curation of an empirical literature base allowing clear comparisons of results across studies, education to broadly disseminate knowledge of the consequences of blast exposure to both clinicians and researchers, and the development of empirically supported and well-validated measures to assess and characterize blast exposure.

## Data availability statement

The raw data supporting the conclusions of this article will be made available by the authors, upon reasonable request.

## Ethics statement

The studies involving humans were approved by Salisbury VA Healthcare System Institutional Review Board. The studies were conducted in accordance with the local legislation and institutional requirements. The participants provided their written informed consent to participate in this study.

## Author contributions

JR: Conceptualization, Data curation, Formal analysis, Investigation, Methodology, Project administration, Resources, Supervision, Writing – original draft, Writing – review & editing. SM: Conceptualization, Data curation, Investigation, Methodology, Project administration, Resources, Supervision, Writing – original draft, Writing – review & editing.
